# Neuroendocrine Disturbances after Brain Damage: An Important and Often Undiagnosed Disorder

**DOI:** 10.3390/jcm4050847

**Published:** 2015-04-28

**Authors:** Fatih Tanriverdi, Fahrettin Kelestimur

**Affiliations:** Department of Endocrinology, Erciyes University Medical School, 38039, Kayseri, Turkey; E-Mail: fatihtan@erciyes.edu.tr

**Keywords:** hypopituitarism, trauma, pituitary, injury

## Abstract

Traumatic brain injury (TBI) is a common and significant public health problem all over the world. Until recently, TBI has been recognized as an uncommon cause of hypopituitarism. The studies conducted during the last 15 years revealed that TBI is a serious cause of hypopituitarism. Although the underlying pathophysiology has not yet been fully clarified, new data indicate that genetic predisposition, autoimmunity and neuroinflammatory changes may play a role in the development of hypopituitarism. Combative sports, including boxing and kickboxing, both of which are characterized by chronic repetitive head trauma, have been shown as new causes of neuroendocrine abnormalities, mainly hypopituitarism, for the first time during the last 10 years. Most patients with TBI-induced pituitary dysfunction remain undiagnosed and untreated because of the non-specific and subtle clinical manifestations of hypopituitarism. Replacement of the deficient hormones, of which GH is the commonest hormone lost, may not only reverse the clinical manifestations and neurocognitive dysfunction, but may also help posttraumatic disabled patients resistant to classical treatment who have undiagnosed hypopituitarism and GH deficiency in particular. Therefore, early diagnosis, which depends on the awareness of TBI as a cause of neuroendocrine abnormalities among the medical community, is crucially important.

## 1. Introduction

Traumatic brain injury (TBI) is a well-known public health problem around the world, and it is associated with increased morbidity, mortality and long-term disability. The mean incidence rate of hospitalized and fatal TBI and the average mortality rate have been reported as 235 per 100,000 and about 15 per 100,000, respectively, in Europe [1]. Winqvist *et al.* reported that by the age of 35, 3.8% of the subjects in their northern Finland birth cohort study had experienced at least one hospitalization for TBI [2]. A huge number of people are seen in emergency departments; the great majority of them, about 235,000 each year, are hospitalized because of non-fatal TBI, and approximately 50,000 die according to reports from the United States [3]. The overall annual incidence of TBI in the United States was reported as 506.4 per 100,000 population [3]. The incidence rate and mortality rate of TBI in India were reported as 344 per 100,000 and 38 per 100,000, respectively [4]. McKinlay *et al.* found that the average incidence of TBI for individuals between 0–25 years of age ranged from 1.10–2.36 per 100 per year, with an overall prevalence of ~30% in a study conducted in Christchurch, New Zealand [5]. Therefore, there is no doubt that TBI is one of the most common causes of mortality and long-term disability among children and adults, young adults in particular. Epidemiological studies estimated that the incidence rate of TBI around the world is much higher than the rate previously reported.

The main causes of TBI are road traffic accidents, falls, violence-related incidents, work injuries, sports-related head traumas, which include hockey, soccer and football, combative sports characterized by chronic repetitive head traumas and war accidents. Road traffic accidents are the leading cause of TBI and account for 50% of all cases; falls and violence-related events are the other most common causes, respectively [6]. The increasing number of cars on the roads and the rise in alcohol consumption among young people, in particular, may be associated with more frequent traffic accidents.

TBI-induced hypopituitarism was first reported about 95 years ago [7]. Until recently, neuroendocrine dysfunction after TBI was recognized as an uncommon abnormality, and only 367 cases of hypopituitarism due to TBI were reported before 2000 [8]. Since then, the relation between TBI and neuroendocrine changes has become one of the hot topics mainly in endocrinology and neurosurgery, and more data exist at the present time.

Recently, sports-related head injury has also been suggested as another cause of hypopituitarism [9]. There are two kinds of sports that may cause TBI: one is combative sports, which are characterized by chronic repetitive brain injury, such as in boxing and kickboxing, in which an athlete may be subjected to head trauma more than 1000 times; the other is non-combative sports, including hockey, swimming and football, which are characterized by single or multiple head traumas during an individual’s sports career [10,11,12,13]. War accidents, which also cause neuroendocrine abnormalities, are heterogeneous in origin and include falls, motor vehicle accidents, shrapnel and bullet wounds and blastic brain injuries [14]. Traffic accidents, falls, sports injuries and war accidents, all of which can result in brain injury, affect a huge number of people and not only cause increased morbidity and mortality, but may also be responsible for hypopituitarism.

## 2. The Frequency of Hypopituitarism after Traumatic Brain Injury

During the last 15 years, several retrospective and prospective studies have revealed that hypopituitarism due to TBI is not rare, and most of the patients had undiagnosed and, so, untreated hypopituitarism [15,16,17,18,19,20,21]. Independent of the severity of head trauma, some degree of hypopituitarism after TBI has been reported in as many as 25%–50% of patients [18,20,21]. Tanriverdi *et al.* reported that 50.9% of the patients with TBI had at least one anterior pituitary hormone deficiency one year after TBI in a prospective study [20]. In a cross-sectional study, 246 patients with moderate to severe TBI included in a German multi-center screening program were evaluated in terms of prevalence of anterior pituitary dysfunction. Hormonal investigation was carried out at an average of 12 ± 8 months (range 4–47 months) after TBI. Hypopituitarism was diagnosed in 21% of the patients, and the frequency of total, partial and isolated hypopituitarism was 1%, 2% and 18%, respectively [22]. Schneider *et al.* analyzed 10 studies including 809 patients and reported the prevalence of any pituitary deficiency as 27.5% [23]. The prevalence of hypopituitarism due to TBI is lower in children than that in adults [24]. The lowest frequency of hypopituitarism was reported by Klose *et al.* [25]. Only 1% of the patients had growth hormone deficiency (GHD) according to tests. However, there are some limitations in that study, which did not include severe TBI patients. The authors used pyridostigmine-growth hormone releasing hormone (GHRH) to confirm the results obtained by insulin tolerance test (ITT), which is the gold standard test. The confirmatory test, namely the pyridostigmine (PD)-GHRH test, is not a commonly-used and well-standardized test. Nevertheless, the study by Klose *et al.* has two useful advantages: a stringent study protocol and the inclusion of a control group. When the extremely high number of people who suffered from TBI is taken into account, even low rates of hypopituitarism, such as 1%, translate into a great number of patients with TBI-induced pituitary dysfunction.

The frequency of pituitary dysfunction varies between the studies, and it depends on factors, including the severity of trauma, type of trauma, time elapsed since the trauma, study population, the design of the study, endocrine tests and the criteria used for the diagnosis of anterior pituitary hormone deficiencies [26]. Pituitary dysfunction was reported to be more common in patients with severe TBI than in those with less severe TBI [15,23].

Concussion is a well-known injury associated with sports, particularly combative sports, including boxing, football and ice-hockey. Although the relation between sports injuries and brain trauma has been established for a long time, pituitary functions in combative sports have not been investigated until recently. Therefore, data regarding the frequency of hypopituitarism due to sports-related brain injury are limited. In a study including actively competing and retired male boxers, 45% of the boxers were found to have growth hormone (GH) deficiency [10]. The same authors reported that 22.7% and 9.1% of amateur kickboxers had GH and adrenocorticotropic hormone (ACTH) deficiency, respectively [11]. In a study including a larger number of actively competing and retired boxers, they found that 15% had GH and 8% had ACTH deficiency. Interestingly, approximately 50% of the retired boxers had GH deficiency, and retired boxers with GH deficiency had significantly lower pituitary volume than retired boxers with normal GH levels [12]. Another kind of sport that may cause brain injury is football. Kelly *et al.* reported that 23.5% of retired football players with a relatively low quality of life had hypopituitarism [13]. Recently, an unusual cause of pituitary dysfunction related to TBI, namely war accidents, has become a new research area. Hypopituitarism and GH deficiency, in particular, were reported as 25% and 42% among victims of combat-related traumatic brain injury, including blast-related mild-TBI [14,27].

## 3. Clinical Manifestation of TBI-Induced Hypopituitarism 

The clinical picture depends on the severity of hypopituitarism and the number of deficient anterior pituitary hormones. The clinical presentation seen in hypopituitarism varies from very subtle findings, which are often not discovered unless a very careful medical history and physical examination have been undertaken, to life-threatening conditions in which patients are seen in the emergency department because of severe manifestations of pituitary failure, such as adrenal crisis, severe hypotension, hypoglycemia and hypothyroidism. Because the mild/subtle manifestations due to hypopituitarism could result from different causes, most patients with hypopituitarism remain undiagnosed and thereby untreated. For example, in a recent study, 52.6% of the patients with hypopituitarism due to Sheehan’s syndrome had non-specific complaints at presentation [28]. 

The data regarding the natural history of TBI-induced hypopituitarism are extremely limited. In a prospective study covering five years, GH deficiency was found to be the most common pituitary hormone deficit at one, three and five years following TBI. It was also reported that most of the pituitary hormone deficiencies improved over five years, but a substantial number of patients still had hypopituitarism at the fifth year. Some patients, although rarely, may develop new onset hypopituitarism or pituitary dysfunction may worsen over the years [20,29,30].

The most common cause of hypopituitarism is pituitary adenoma, and not uncommonly, patients with pituitary adenoma seek medical advice because of findings due to mass effects, but not for findings of hormonal abnormalities. In contrast, in patients with TBI-induced hypopituitarism, the presenting symptoms are often related to deficient anterior pituitary hormones only. Thus, the clinical picture in patients with hypopituitarism due to TBI is apparently related to the severity of pituitary hormone deficiencies. Current data suggest that isolated GH deficiency is probably the most common anterior pituitary hormone deficiency [10,18,30]. In a Pfizer International Metabolic database (KIMS)-based observational study, Casanueva *et al.* compared 51 patients with GHD due to TBI with 688 patients with GHD due to non-functional pituitary adenoma (NFPA). TBI-induced GHD patients were significantly shorter than the patients with NFPA-induced GHD. The patients had not been treated with GH before being included in KIMS [31]. The results of an analysis of the German Pfizer International Metabolic database, including patients with TBI-induced hypopituitarism, showed that patients with childhood onset GH deficiency were significantly shorter than adult onset GH-deficient patients. The IGF-I SDS levels at baseline were also significantly lower in patients with childhood onset TBI than in adult onset TBI patients [32]. 

Schneider *et al.* attempted to understand the predictors of anterior pituitary insufficiency after traumatic brain injury (TBI) in 78 patients. The patients were investigated in the first three months ± two weeks after TBI and reinvestigated 12 months ± four weeks after TBI. Pituitary functions were assessed by basal hormone levels and dynamic tests, including GHRH + arginine and 250 µg ACTH stimulation tests. In addition, the patients had initial and follow-up computed tomography (CT) scans. MRI was also performed in equivocal cases. Among the clinicoradiological parameters, diffuse axonal injury and basal skull fracture were found to be associated with increased prevalence of TBI-induced hypopituitarism [33]. Furthermore, hypopituitarism due to TBI is associated with cognitive dysfunction revealed by a decreased P300 amplitude, which is related to the updating of working memory content and attention [34]. Deficits in simple attention, increased reaction time and greater emotional disruption were reported in patients with isolated GH deficiency due to TBI, but not in patients with TBI who have normal pituitary functions [35]. It seems that cognitive dysfunction seen after TBI is not only the result of the brain injury itself, but also of hypopituitarism. GH deficiency, in particular, may result in cognitive abnormalities. Ioachimescu *et al.* found that GH deficiency has adverse effects on executive abilities and mood in male veterans with mild TBI [14]. Prodam *et al.* in a cross-sectional retrospective study showed that patients with TBI-induced hypopituitarism had a worse metabolic profile, including insulin resistance, altered glucose levels and dyslipidemia than TBI victims who had normal pituitary functions [36]. 

Since the importance of hypopituitarism after TBI has only been recognized recently, the medical community was previously totally unaware that TBI could be a significant cause of hypopituitarism, and a history of TBI was not included among the questions aimed at clarifying the underlying cause of hypopituitarism. Unawareness of TBI-induced hypopituitarism is one of the most likely reasons responsible for ignoring the problem and leaving the patient undiagnosed and thereby untreated. It was reported that the most suitable time for endocrine assessment is one year after TBI, and the best TBI victims for screening are those with moderate or severe TBI or if mild TBI is associated with conditions suggesting a worse prognosis [37].

## 4. The Pathophysiology of TBI-Induced Hypopituitarism

The pathophysiology of pituitary dysfunction after TBI is not yet fully understood despite several experimental and clinical studies. It seems that hypopituitarism occurs after TBI because of an imbalance between neurodegenerative and neuroprotective mechanisms. Direct mechanical trauma, vascular insult, hypothalamic damage, inflammatory changes, compression from hemorrhage, edema or increased intracranial pressure, genetic predisposition and autoimmunity may play a role in the development of neuroendocrine abnormalities [38,39,40]. Autopsy studies revealed that the pituitary stalk, anterior and posterior pituitary may be affected after TBI, and hemorrhage, necrosis and fibrosis are the most commonly seen lesions [41]. In terms of anatomical localization, the hypothalamus, stalk and pituitary gland are vulnerable to direct mechanical trauma, ischemic changes and compression due to edema. Long portal vessels, which take blood supply from the superior hypophyseal artery, pass through the diaphragm sella and may be damaged after head trauma more easily. Since somatotroph cells are perfused by the long hypophyseal portal system, it is not surprising that GH is the most common deficient hormone after TBI. Since lower pituitary dysfunction was reported in TBI victims who had the APO E3/E3 genotype, genetic predisposition may be an interesting factor in the development of hypopituitarism [40]. Recent studies revealed that autoimmunity may have some role in the development and/or worsening of hypopituitarism. More commonly, hypopituitarism was found in patients with TBI and athletes who had higher antipituitary and antihypothalamic antibodies [39,42].

TBI may be chronically repetitive as seen in boxing and kickboxing, in which the athlete may be subjected to up to 3000 blows to the head, or it may be characterized by a single or several head traumas, as seen in road accident or falls. No study has compared these two types of TBI. In contact sports, including boxing, kickboxing and football, the most common type of head trauma is concussion, and the cumulative impacts are responsible for chronic traumatic brain injury [9]. Interestingly, only positivity for antihypothalamus antibodies (AHAs), but not for antipituitary antibodies (APAs) has been found to be associated with hypopituitarism in boxers; this implies that hypothalamic damage may be more important than pituitary damage in athletes participating in contact sports in contrast to TBI due to other causes, in which both hypothalamic and/or pituitary damage may account for pituitary dysfunction [39,40,41,42].

Since hypopituitarism has been reported as more common in patients with moderate (Glascow Coma Scale (GCS) [8,9,10,11,12]) and severe (GCS <8) TBI or at least suggested by some studies [15,16,20,21]; these patients should be screened, except for patients with severe disability due to severe TBI in whom hormonal replacement therapy is not beneficial. Testing of all mild TBI patients (GCS >13) is not cost effective. Current data suggest that patients with mild TBI who require hospitalization for at least 24 hours, who have radiological abnormalities on initial CT and who have manifestations of hypopituitarism, should be screened at any time after TBI [43]. Screening the pituitary function after TBI is recommended for at least five years according to the current data [20,29,30]. As mentioned previously, different tests and assays for hormone measurement have been used in different studies carried out in various countries. The lack of standardized diagnostic criteria for GH deficiency, in particular, and the different GH assays used in various studies make the determination of GH deficiency problematic and very different. For this reason, the evidence-based data on the diagnosis of GH deficiency and thereby our recommendations are eminence- rather than evidence-based.

## 5. Treatment of Hypopituitarism in Patients with TBI

The main principle in the treatment of hypopituitarism due to TBI is the appropriate replacement of deficient hormone(s), if indications exist. There are no data as to whether treatment in patients with TBI-induced hypopituitarism differ from patients with hypopituitarism due to non-TBI causes. Since the most common deficient hormone is GH and isolated GH deficiency is not uncommon, most of the studies published so far in the literature are related to the effects of GH replacement. Recombinant human growth hormone (rhGH) replacement therapy improves muscle force production, body composition and aerobic capacity [44]. In a retrospective study, 84 patients with TBI who were included in the German KIMS database were compared with 84 patients with non-functioning pituitary adenoma, and it was shown that quality of life (QoL) was significantly improved in both groups after GH replacement therapy [32]. GH replacement therapy not only improves QoL in patients with TBI-induced hypopituitarism, but also improves metabolic abnormalities, including glucose intolerance and abnormal lipid profile. In a recent study, Gardner *et al.* reported that QoL was worse in TBI patients with GHD when compared to NFPA patients, and one-year improvement in QoL after GH replacement therapy was greater in TBI patients [45]. 

In the acute phase of TBI, only glucocorticoid replacement therapy is required in patients with ACTH deficiency, which is life-threatening. Replacement of GH, testosterone/estradiol and thyroid hormone is not recommended in the acute phase of TBI, because the hormonal changes in the early period after TBI are the physiological response to critical illness [23,46,47,48]. On the other hand, hyponatremia may occur during the acute phase, and the most frequent cause is the syndrome of inappropriate antidiuretic hormone secretion (SIADH), which was shown to be transient in all cases [47]. Patients with SIADH after TBI were found to be glucocorticoid deficient, and hyponatremia was responsive to glucocorticoid treatment [49]. Therefore, in the case of hyponatremia after TBI, SIADH should be taken into account, and ACTH deficiency should be screened and treated if it is diagnosed.

In conclusion, TBI is a common public health problem worldwide, and it is associated with increased morbidity and mortality. In recent years, neuroendocrine changes, including GH deficiency in particular, have been recognized as a significant consequence of TBI. The real prevalence of TBI-induced hypopituitarism is underestimated, probably due to unawareness of the problem. The subtle clinical manifestations of mild hypopituitarism following TBI mean that patients are undiagnosed and thereby untreated. Doctors, and especially endocrinologists, should be aware of this often unnoticed health problem.
